# Second‐Generation Epigenetic Clocks and Socioeconomic Status Predict Midlife Cognitive Status

**DOI:** 10.1002/alz.093357

**Published:** 2025-01-03

**Authors:** Sophie A. Bell, Christopher R. Beam, Deborah Finkel, A. Cevelle Barna, Ariel King, Kendra Sikes, Lesa Ryan, Deborah Winders Davis, Eric Turkheimer

**Affiliations:** ^1^ University of Virginia, Charlottesville, VA USA; ^2^ University of Southern California, Los Angeles, CA USA; ^3^ Jönköping University, Jönköping Sweden; ^4^ Norton Children’s Research Institute, Louisville, KY USA; ^5^ University of Louisville, Louisville, KY USA

## Abstract

**Background:**

DNA methylation (DNAm) age measures, or ‘epigenetic clocks’, surpass chronological age in their ability to predict age‐related morbidities and mortality. The Louisville Twin Study (LTS) presents an opportunity to clarify the role of early life environmental exposures and development in biological and cognitive aging in midlife. We expect that second‐generation DNAm age measures trained to predict age related outcomes and death, independent of chronological age, will be sensitive to cognitive ability in midlife.

**Method:**

Data collection of the midlife phase of the LTS is ongoing. This study utilizes data from 281 middle‐aged twins, including 56 monozygotic and 41 dizygotic complete twin pairs (mean age 51.9 years ± 7.03). Midlife cognitive ability (IQ) was measured using the Wechsler Adult Intelligence Scale Fourth Edition. DNAm age acceleration was calculated using five clock algorithms: Horvath 1, Horvath 2, Hannum, GrimAge, and PhenoAge. We conducted an exploratory factor analysis to identify common factors as latent variables among the five clocks. Composite scores for factors were calculated, and genetically informed, quasi‐causal regression models were fitted in which adult IQ was predicted from childhood socioeconomic status (SES), DNAm factor composite score, and the interaction between DNAm age and SES.

**Result:**

Factor analysis produced a two‐factor structure: first‐generation clocks (Horvath 1, Horvath 2, and Hannum) and second‐generation clocks (PhenoAge and GrimAge). As predicted, childhood IQ and childhood SES predict adult IQ. Acceleration in the second‐generation factor predicts lower midlife cognitive ability (*b* = −0.15, *SE* = 0.068, *p* <.05). Additionally, there is an interaction in the second‐generation model such that people raised in higher SES families show a less negative relationship between DNAm age acceleration and adult IQ (*b* = 0.092, *SE* = 0.036, *p* <.05). However, there is no significant effect of the first‐generation factor on adult IQ, and no SES interaction effect.

**Conclusion:**

Second‐generation DNAm age measures show promise in their predictive value for cognitive aging processes. Moreover, the longitudinal twin design of the LTS serves as a quasi‐experimental framework to investigate hypothesized causal effects of DNAm on cognitive aging. Finally, our findings related to childhood SES suggest that the epigenome is malleable and responsive to early‐life stressors.

